# Prediction of new-onset atrial fibrillation with the C_2_HEST score in patients admitted with community-acquired pneumonia

**DOI:** 10.1007/s15010-024-02286-x

**Published:** 2024-05-03

**Authors:** Daniele Pastori, Danilo Menichelli, Giulio Francesco Romiti, Angela Pia Speziale, Pasquale Pignatelli, Stefania Basili, Francesco Violi, Roberto Cangemi

**Affiliations:** 1 Department of Clinical Internal, Anesthesiological and Cardiovascular Sciences, Sapienza University of Rome, Rome, Italy; 2 Department of General Surgery, Surgical Specialties and Organ Transplantation “Paride Stefanini”, Sapienza University of Rome, Rome, Italy; 3 Department of Translational and Precision Medicine, Sapienza University of Rome, Rome, Italy

**Keywords:** Community-acquired pneumonia, C_2_HEST score, Atrial fibrillation, New-onset AF, CAP

## Abstract

**Purpose:**

Patients hospitalized for community-acquired pneumonia (CAP) may have a higher risk of new-onset atrial fibrillation (NOAF). The C_2_HEST score was developed to evaluate the NOAF risk in the general population. Data on the value of the C_2_HEST score in acute patients admitted with CAP are lacking. We want to establish the predictive value of C_2_HEST score for NOAF in patients with CAP.

**Methods:**

Patients with CAP enrolled in the SIXTUS cohort were enrolled**.** C_2_HEST score was calculated at baseline. In-hospital NOAF was recorded. Receiver-operating Characteristic (ROC) curve and multivariable Cox proportional hazard regression analysis were performed.

**Results:**

We enrolled 473 patients (36% women, mean age 70.6 ± 16.5 years), and 54 NOAF occurred. Patients with NOAF were elderly, more frequently affected by hypertension, heart failure, previous stroke/transient ischemic attack, peripheral artery disease and hyperthyroidism. NOAF patients had also higher CURB-65, PSI class and CHA_2_DS_2_-VASc score. The C-index of C_2_HEST score for NOAF was 0.747 (95% confidence interval [95%CI] 0.705–0.786), higher compared to CURB-65 (0.611, 95%CI 0.566–0.655, p = 0.0016), PSI (0.665, 95%CI 0.621–0.708, p = 0.0199) and CHA_2_DS_2_-VASc score (0.696, 95%CI 0.652–0.737, p = 0.0762). The best combination of sensitivity (67%) and specificity (70%) was observed with a C_2_HEST score ≥ 4. This result was confirmed by the multivariable Cox analysis (Hazard Ratio [HR] for C_2_HEST score ≥ 4 was 10.7, 95%CI 2.0–57.9; p = 0.006), independently from the severity of pneumonia.

**Conclusion:**

The C_2_HEST score was a useful predictive tool to identify patients at higher risk for NOAF during hospitalization for CAP.

**Clinical Trial Registration:**

www.clinicaltrials.gov (NCT01773863)

## Introduction

Community-acquired pneumonia (CAP) is a common infectious disease with an incidence estimated between 1 and 25 cases per 1000 inhabitants per year [1] The incidence of CAP is higher in males, in patients with immunodeficiency and/or with comorbidities such as chronic obstructive pulmonary disease (COPD) [1, 2]. While mortality rates in patients with CAP have decreased over recent decades [3], CAP continues to be the predominant infectious disease requiring hospitalization and remains the primary cause of mortality among patients with infectious disease.

Patients with CAP, especially those who require hospitalization, may develop cardiovascular complications, such as acute heart failure (HF), acute coronary syndrome and arrhythmias [4, 5]. Among supraventricular arrhythmias, atrial fibrillation (AF) is commonly associated with CAP as showed by a retrospective study including 4408 patients with CAP, of whom 9.3% had a new-onset AF (NOAF) [6]. This proportion of patients with NOAF is coherent with a prospective multicenter study performed on 1182 patients hospitalized for CAP [7], in which a proportion of 9.2% of patients with a new episode of AF was observed. Importantly, NOAF has also been associated with higher risk of in-hospital mortality in patients hospitalized for CAP, as shown by a retrospective study performed on 519,750 patients with CAP, in whom AF diagnosed during hospital admission considerably associated the risk of in-hospital mortality (23.84% vs. 12.24%, p < 0.001) [8].

Previous studies emphasize that NOAF is a feature of severe CAP and often occurs in the early phase of pneumonia; furthermore, in 40% of cases, patients do not revert to sinus rhythm but may develop persistent AF beyond the infection itself [9]. Early detection of AF is of clinical relevance as it can exacerbate cardiac dysfunction in CAP patients, who are already at heightened risk of cardiovascular events [7], and for the increased risk of thromboembolic stroke [10, 11]. Thus, patients at increased risk of NOAF should be carefully monitored to prevent and to promptly treat AF. However, so far clinical characteristics of patients developing NOAF are not well defined, and there is no validated strategy to flag up patients at higher risk of NOAF during CAP.

Recently, a simple clinical score was developed and validated to predict NOAF in large samples of general population from China and Korea [12]. According to the original study [12], the C_2_HEST score includes six easily available clinical variables such as coronary artery disease (CAD, 1 point), COPD, (1 point), arterial hypertension (1 point) and hyperthyroidism (1 point), while age ≥ 75 years and systolic HF scored 2 points each. This score was also validated in Western countries in several clinical settings. Indeed, in a nationwide French study performed on 240,459 post-ischemic stroke patients, the C_2_HEST score showed a good predictive value suggesting that it may be potentially used as a risk stratification tool to detect post-stroke AF [13]. Furthermore, in a selected cohort of 189 patients undergoing catheter ablation, the C_2_HEST score showed a good predictive value to predict recurrences of AF (area under curve [AUC] 0.769) [14]. Finally, a recent study performed on 555 patients from the REALE-ACS registry [15], that enrolled patients undergoing percutaneous coronary intervention for acute coronary syndrome, confirmed a notable predictive value of this score in this clinical setting (AUC 0.71, 95% confidence interval [95%CI] 0.67–0.74) [15].

However, there were no study that evaluated CH_2_EST to predict NOAF in acute patients admitted with CAP.

Based on this, the aim of our study was to evaluate the predictive value of the C_2_HEST score against NOAF in patients with hospitalized for CAP in the SIXTUS study.

## Methods

We enrolled consecutive patients from 2015 to 2019 admitted to Policlinico Umberto I of Rome with diagnosis of CAP and then a prospective follow-up was performed. All patients gave written informed consent. The study was conducted according to the principles stated in the Declaration of Helsinki and was approved by the local ethics committee. The study has been registered on www.clinicaltrials.gov (NCT01773863).

### Inclusion and exclusion criteria

We included all consecutive patients aged at least 18 years with clinical presentation of an acute illness with at least two or more of the signs or symptoms of CAP, as reported in a previous study [7] and the presence of new consolidation(s) on a chest radiograph [16]. CAP diagnosis was defined if it did not fulfil the criteria for healthcare-associated or hospital-acquired pneumonia [17]. Exclusion criteria included radiographic evidence of preexisting infiltrates; immunosuppression (human immunodeficiency virus infection, chemotherapy, high dose of immunosuppressive agents); critical illness requiring admission to an intensive care unit, presence of malignancy; pregnancy, or breastfeeding; documented severe allergy to antibiotics; and healthcare-associated pneumonia [17]. Patients with paroxysmal, persistent or permanent AF were excluded for this study.

### Baseline assessment

Demographic characteristics and comorbidities for each patient were collected at baseline at hospital admission. Clinical history of arterial hypertension, diabetes mellitus, COPD, dyslipidaemia, previous CAD, peripheral artery disease (PAD), HF, were collected. These comorbidities were defined as previously reported [18]. Baseline pharmacological therapy was assessed. In-hospital treatment decisions were made on the basis of the managing clinical physician’s judgment. In enrolled patients, in-hospital blood laboratory tests and 12-lead electrocardiogram were collected.

Then, we calculated the PSI and CURB-65 score to define pneumonia severity [19, 20]. Finally, also CHA_2_DS_2_-VASc score [21], a common score used to estimate the thromboembolic risk of AF and recently proposed also as tool to predict the risk of NOAF [22] was calculated.

### Definition of C_2_HEST score

As previous reported [12], C_2_HEST score is defined by six items: 1 point for CAD, 1 point for COPD, 1 point for arterial hypertension and 1 point for hyperthyroidism, while 2 points were counted for age ≥ 75 years and systolic HF. the sum of all items can reach a maximum of 8 points.

### Definition of clinical outcome

Clinical outcome of our study is NOAF. It is defined as a newly recognized episode of AF, developed during hospitalization, in subjects who were in sinus rhythm upon admission to the hospital, and documented by medical records as electrocardiogram, pacemaker or implantable cardioverter defibrillator, loop recorder or dynamic 12-lead continuous electrocardiogram (ECG). In our study, a 12-lead ECG was performed on each recruited patient upon admission and repeated every 24 h during hospitalization. If patients presented symptoms attributable to atrial fibrillation, additional 12-lead electrocardiograms were performed. A 12-lead ECG with more than 30 s of atrial fibrillation was considered as NOAF. Adjudication of AF and AF treatment strategies were conducted by cardiologists who did not participate in patient recruitment and follow-up, in accordance with the ESC guidelines [23].

### Statistical analysis

Continuous variables were expressed as mean and standard deviation or as median and interquartile range (IQR). Categorical variables were expressed as percentages. Student *t*-test was used to compare means. Pearson χ^2^ test was used to compare proportions. Group comparisons were made with analysis of variance (ANOVA).

A first descriptive analysis was performed to report clinical characteristics of patients developing NOAF.

To evaluate the predictive value of C_2_HEST score, we performed a “Receiver-operating Characteristic” (ROC) curve, estimating the AUC to establish the predictive role of the score. Then, we compared the ROC curves of C_2_HEST, CURB-65, PSI and CHA_2_DS_2_-VASc score to identify the best score to predict NOAF in patients hospitalized for CAP. Furthermore, we performed a multivariable Cox analysis to identify clinical factors associated with NOAF.

Only p-values less than 0.05 were regarded as statistically significant. All tests were two tailed, and analyses were performed using computer software packages (IBM SPSS Statistics version 23.0).

## Results

After excluding 77 patients with AF at baseline, 473 patients with CAP were included. Of whom, 36% were women and mean age was 70.6 ± 16.5 years. Clinical characteristics of the cohort are reported in Table 1. During in-hospital admission, 54 patients (11%) had a NOAF. Clinical characteristics of patients according to NOAF were reported in Table 1. Patients with NOAF were elderly and more frequently affected by arterial hypertension, HF, a previous history of stroke/transient ischemic attack (TIA), PAD, and hyperthyroidism. Moreover, patients who developed AF had a lower left ventricular ejection fraction (LVEF) and a higher left atrium diameter (LAD). Furthermore, patients with NOAF had a higher CURB-65, PSI and CHA_2_-DS_2_-VASc scores (Table 1).

**Table 1:** Clinical characteristics of patients according to new-onset atrial fibrillation

	Total (n: 473)	Patients without AF (n: 419)	Patients with NOAF (n: 54)	p-value
Mean age (years)	70.6 ± 16.5	69.4 ± 16.9	79.9 ± 9.6	< 0.001
Men (%)	301 (64)	263 (63)	38 (70)	0.274
Age ≥ 75 years (%)	237 (50)	194 (46)	43 (80)	< 0.001
Body mass index (mean)	26.2 ± 6.2	26.2 ± 6.2	25.9 ± 3.9	0.711
Arterial hypertension (%)	320 (68)	274 (65)	46 (85)	0.003
Diabetes mellitus (%)	110 (23)	96 (23)	15 (26)	0.622
COPD (%)	139 (29)	121 (29)	18 (33)	0.499
Heart failure (%)	79 (17)	58 (14)	21 (39)	< 0.001
Coronary heart disease (%)	127 (27)	104 (25)	23 (43)	0.006
History of stroke/TIA (%)	48 (10)	38 (9)	10 (18)	0.030
Chronic kidney disease (%)	70 (15)	61 (15)	9 (17)	0.681
Peripheral artery disease (%)	30 (6)	21 (5)	9 (17)	< 0.001
Hyperthyroidism (%)	4 (1)	2 (1)	2 (4)	0.015
LVEF (%)	53.0 ± 10.0	53.7 ± 9.6	47.9 ± 11.4	< 0.001
LAD, mm	39.5 ± 6.1	38.9 ± 6.1	43.8 ± 5.0	< 0.001
Left atrium area, cm^2^	19.2 ± 6.9	18.5 ± 6.6	24.7 ± 6.4	< 0.001
Antiplatelets (%)	188 (40)	163 (39)	25 (46)	0.296
In-hospital admission length (days)	10 [7−13]	9 [6−12]	12 [8−16]	< 0.001
Statins (%)	147 (31)	128 (31)	19 (36)	0.407
PSI class (mean)	3.27 ± 1.02	3.20 ± 1.01	3.85 ± 0.91	< 0.001
CURB-65 score (mean)	1.53 ± 0.93	1.50 ± 0.85	1.78 ± 0.71	0.013
CHA_2_DS_2_-VASc score (mean)	2.47 ± 1.70	2.34 ± 1.68	3.48 ± 1.46	< 0.001
C_2_HEST score (mean)	2.60 ± 1.90	2.40 ± 1.90	4.00 ± 1.40	< 0.001

*AF* atrial fibrillation, *COPD* chronic obstructive pulmonary disease, *LAD* left atrium diameter, *LVEF* left ventricular ejection fraction, *NOAF* new-onset atrial fibrillation, *TIA* transient ischemic attack

### Predictive value of C_2_HEST to predict NOAF

**Fig. 1:**
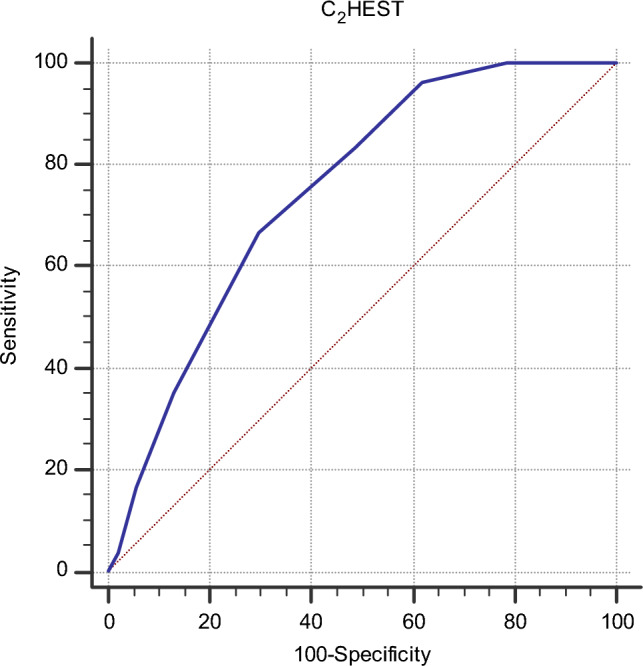
Receiver operating characteristic (ROC) curves of C_2_HEST score in predicting new-onset atrial fibrillation

Mean C_2_HEST score was 2.6 ± 1.9. Patients with NOAF had higher C_2_HEST score compared to without ones (4.0 ± 1.4 vs. 2.4 ± 1.9, respectively, p < 0.001). Using ROC curves, the C-index of C_2_HEST score to predict NOAF is 0.747 (95%CI 0.705–0.786) (Fig. 1). The sensitivity and specificity of each point of C_2_HEST score were reported in Table 2. A high sensitivity and low specificity were observed in low C_2_HEST score points (0–3), with a progressive increase of specificity and decrease of sensitivity according to an increase of C_2_HEST score. The best combination of sensitivity (67%) and specificity (70%) was observed with a C_2_HEST score ≥ 4 (Table 2).

**Table 2:** Sensitivity and specificity according to each point of C_2_HEST score

Criterion	Sensitivity	95%CI	Specificity	95%CI
≥ 0	100.00	93.4−100.0	0.00	0.0−0.9
> 0	100.00	93.4−100.0	21.48	17.6−25.7
> 1	96.30	87.3−99.5	37.95	33.3−42.8
> 2	83.33	70.7−92.1	51.31	46.4−56.2
> 3	66.67	52.5−78.9	70.17	65.5−74.5
> 4	35.19	22.7−49.4	87.11	83.5−90.2
> 5	16.67	7.9−29.3	94.51	91.9−96.5
> 6	3.70	0.5−12.7	97.85	96.0−99.0
> 7	0.00	0.0−6.6	100.00	99.1−100.0

*95%CI* 95% confidence interval

**Fig. 2:**
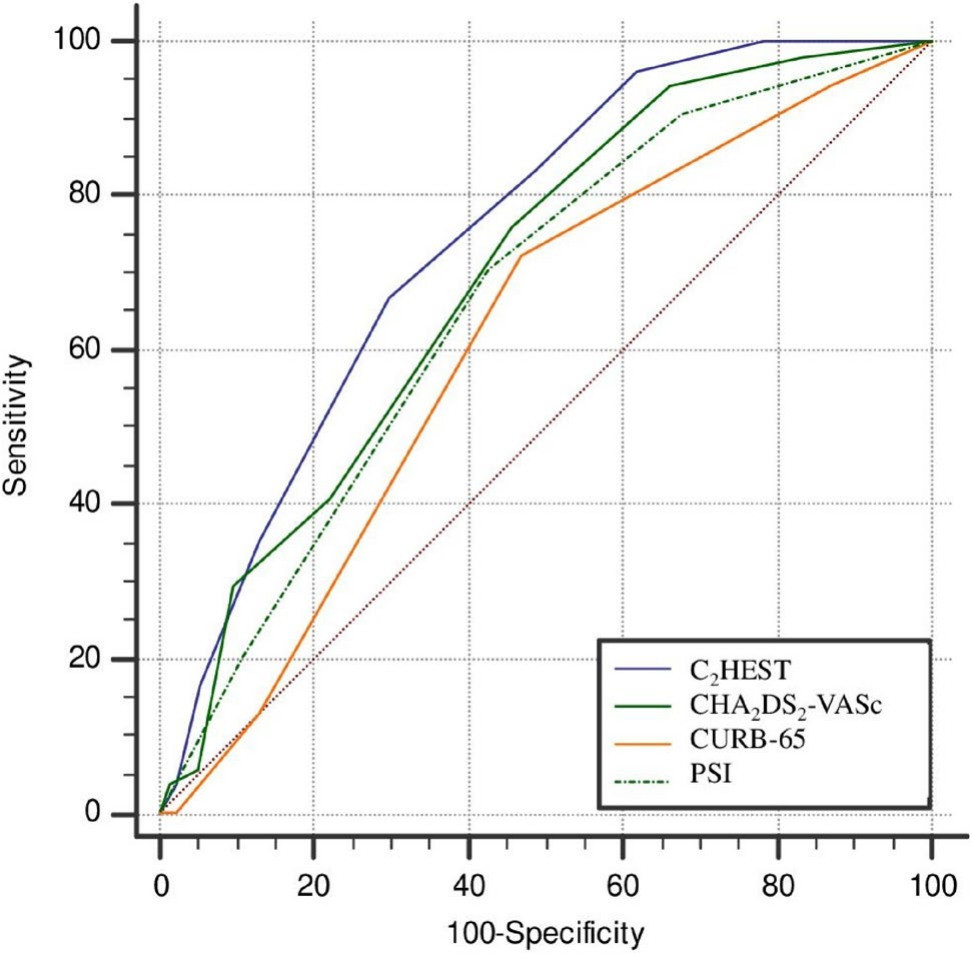
Comparison of receiver operating characteristic (ROC) curves of C_2_HEST, CURB-65, PSI and CHA_2_DS_2_-VASc scores in predicting new-onset atrial fibrillation

The C_2_HEST score showed a higher predictive value for NOAF compared to CURB-65 (C-index 0.611; 95%CI 0.566−0.655, p = 0.0016 for comparison), PSI (C-index 0.665; 95%CI 0.621–0.708, p = 0.0199 for comparison) and to the CHA_2_DS_2_-VASc score (C-index 0.696; 95%CI 0.652–0.737, p = 0.0762, for comparison) (Fig. 2).

**Fig. 3:**
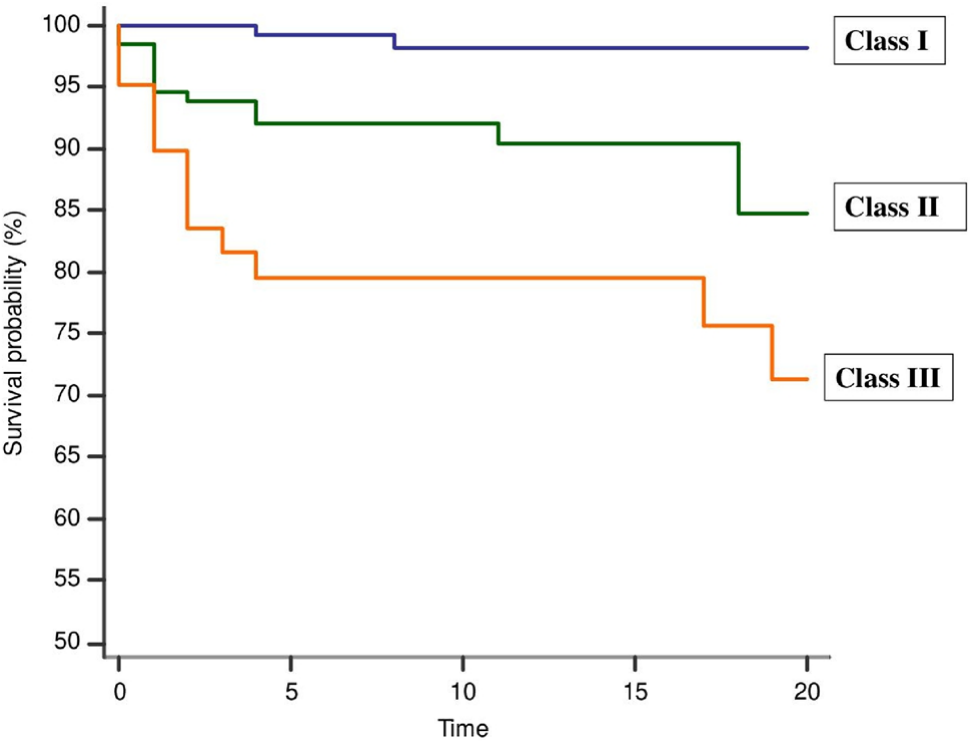
Time to new-onset atrial fibrillation according to C_2_HEST score classes (Class I: 0–1 point, Class II: 2–3 points, Class III: ≥ 4 points)

Furthermore, we classified the risk of NOAF according to C_2_HEST score in three classes: patients with low risk (0–1 point, 34%, n = 161); medium risk (2–3 points, 32%, n = 151); and high risk (≥ 4 points, 34%, n = 161) (Table 3). A higher class of C_2_HEST score was associated with a lower time to NOAF during in-hospital follow-up (median 8 days, interquartile range [IQR] 3.5–13 days) as showed in Fig. 3.

**Table 3:** Risk of new-onset atrial fibrillation according to three C_2_HEST risk groups

C_2_HEST	No. of patients	No. of NOAF	Incident rate (%)	Odds ratio [95%CI]	p-value
0–1	161	2	1.0	Ref	
2–3	151	16	11.0	9.4 [2.1–41.7]	0.003
≥ 4	161	36	22.0	22.9[5.4–96.9]	< 0.001

*95%CI* 95% confidence interval, *NOAF* new onset atrial fibrillation

Then, we performed a multivariable Cox analysis that showed a ten-fold and five-fold higher risk to develop AF during CAP in patients with C_2_HEST ≥ 4 points (Hazard Ratio [HR] 10.7; 95%CI: 2.0 – 57.9; p = 0.006) and C_2_HEST of 2–3 points (HR 5.3; 95%CI 1.1 −26.7; p = 0.043), respectively, compared to patients with C_2_HEST of 0–1 point, independently from CAP severity, estimated by the PSI Score. Comparable outcomes were observed after adjusting for the CURB-65 score, the CHA2DS2-VASc score, additional comorbidities not encompassed in the C2HEST score, as well as age (years) and sex (Table 4).

**Table 4:** C_2_HEST classes as predictors of NOAF: multivariable COX regression analyses

	*HR*	*95% CI*		*P*
Model A				
High vs. low risk C_2_HEST class	10.7	2.0	57.9	0.006
Medium vs. low risk C_2_HEST class	5.3	1.1	26.7	0.043
Model B				
High vs. low risk C_2_HEST class	19.1	4.3	84.4	< 0.001
Medium vs. low risk C_2_HEST class	8.4	1.8	38.6	0.006
Model C				
High vs. low risk C_2_HEST class	15.1	3.0	76.9	0.001
Medium vs. low risk C_2_HEST class	7.2	1.5	34.4	0.013
Model D				
High vs. low risk C_2_HEST class	13.7	2.4	78.2	0.003
Medium vs. low risk C_2_HEST class	6.4	1.1	35.8	0.035
Model E				
High vs. low risk C_2_HEST class	11.9	2.0	69.4	0.006
Medium vs. low risk C_2_HEST class	5.9	1.0	33.7	0.046

Model A: after adjusting for pneumonia severity index (PSI) classes, Model B: after adjusting for CURB-65 score; Model C: after adjusting for CHA_2_DS_2_-VASc score; Model D: after adjusting for age, sex, peripheral arterial disease, and diabetes, Model E: after adjusting for age, sex, peripheral arterial disease, diabetes, and PSI

*CI* confidence interval; High risk C_2_HEST class: C_2_HEST score ≥ 4 points; medium risk C_2_HEST class: C_2_HEST score of 2–3 points, low risk C_2_HEST class: C_2_HEST score ≤ 1 point; *HR* hazard ratio

## Discussion

Our study found that the C_2_HEST score was a useful tool to predict NOAF in patients with CAP with an AUC of 0.747. The risk of NOAF gradually increased with the score, and a C_2_HEST score ≥ 4 showed the best sensitivity and specificity. Furthermore, the C_2_HEST score performed better that other commonly used clinical risk scores.

The association between incident AF and CAP is well documented as showed by a previous study performed on 69,776 patients using data from the National Health Insurance Research Database in Taiwan [24]. In this study the incidence rate of AF in patients without pneumonia was 1.2 per 1000 person-months, while it was 4.08-fold higher in those with pneumonia [24].

In our study we found that 11% of patients developed NOAF. This proportion is similar to that reported in our previous multicenter study [7], and in another more recent study [25] performed on 1092 patients with pneumococcal pneumonia that confirmed a high rate of NOAF (9.9%) in patients with this disease. In this study, older age, heavy drinking, respiratory rate ≥ 30/minute, leukopenia, severe inflammation and bacteraemia were independent risk factors for developing NOAF [25].

Although this association has been consistently observed in previous studies, the pathogenesis is still unclear. A potential role in this association may be assumed by Nox2-derived oxidative stress. Elevated Nox2 activity was noted in the atria of patients undergoing cardiac surgery who subsequently developed atrial fibrillation, indicating that oxidative stress may have a significant role in its pathogenesis [26]. In the context of CAP, a study performed on 432 patients showed that patients who experienced NOAF had a more severe disease and an enhanced Nox2-derived oxidative stress compared to those who did not [9]. This study also suggested low-grade endotoxemia, that may have intestinal origin [27], as a potential trigger for oxidative stress production. Indeed, CAP has been associated to dysfunction of the intestinal barrier and subsequent translocation of bacterial products into the systemic circulation [27]. Several factor including metabolic diseases, aging and systemic inflammation may contribute to low-grade endotoxemia, inducing changes in gut microbiota [28]. If this hypothesis were to be validated, targeting the gut microbiota could emerge as a novel therapeutic strategy to mitigate cardiovascular complications, including NOAF in the context of CAP.

The incidence of NOAF was also studied in patients with SARS-COV2-related pneumonia. Indeed, a large cohort study [29] including 3,064 patients hospitalized for COVID-19, showed that 5.4% of patients developed AF during hospitalizations, that was associated with higher risk of death [29].

However, the long-term role of NOAF on patients with CAP is still unclear: indeed, although about 50% of patients with NOAF during CAP experienced a spontaneous or post-cardioversion return to sinus rhythm [9], no strong evidence about recurrence of AF is still available. However, epidemiological data showed a higher risk of AF exacerbations during winter seasons [30]; this may be explained with higher risk of CAP in this period, and we could assume that an history of NOAF during hospitalization for CAP may be harmful for AF recurrence.

We applied the C_2_HEST score to a cohort of prospectively enrolled patients with CAP requiring hospitalization. We found a C-index of C_2_HEST score of 0.747. This figure is similar to those found in previous studies. Indeed, a study performed on 240,459 French post-ischemic stroke found that C_2_HEST score had a C-index of 0.734 in predicting incident AF during 7.9 ± 11.5 months of follow-up [13]. Similar results with a AUC of 0.78 were observed in a cohort of 370,874 patients with rheumatological disease [31] and were coherent with the AUC of 0.75 reported in the cohort of development and internal validation made up of 471,446 subjects from the Chinese Yunnan Insurance Database (internal derivation cohort) [12].

Of note, the C_2_HEST score encompasses comorbidities known to be associated both with NOAF in general population [12]. and with cardiovascular events in CAP [7]. Other predictive tools for incident AF have been proposed, including the ARIC score [32], the FHS score [33], and the CHARGE-AF score [34]. These scoring systems were developed from extensive cohorts and demonstrated fair predictive performance. Nevertheless, these scores necessitate numerous instrumental and laboratory variables for calculation that are not readily available in clinical practice. Conversely, the C_2_HEST score relies solely on the patient’s age and past clinical history, a simplicity that could be clinically relevant, particularly considering that cardiovascular complications such as NOAF typically manifest within the first 24–28 h following pneumonia diagnosis.

### Clinical implications

Our study showed that C_2_HEST score, a simple and clinical tool, may be useful in clinical practice to identify patients with high risk to develop NOAF during CAP. This may be helpful to clinicians to select patients that have benefits from a screening to early detect NOAF so reducing the risk of thromboembolic stroke starting an adequate anticoagulant therapy. It's notable that cardiovascular complications, including NOAF, have been linked to increased short- and long-term mortality risks in this setting [7, 35] and a recent evidences suggests that NOAF itself may increase the mortality risk in CAP patients [8, 36]. Of note, in our study the hospital stay was longer in patients who experienced NOAF. Thus, an early and tight management of precipitating factors may be helpful to prevent NOAF and its complications reducing hospital stay, disability and possibly mortality.

### Limitations

Our study had also some limitations. Firstly, we enrolled only Western patients admitted with CAP, for this reason, our results may be not applicable to other countries. We also did not investigate if pneumonia was of bacterial, viral origin, or both. However, despite current diagnostic tests, no specific pathogen is usually detected in most patients hospitalized for CAP [37]. We did not study NOAF in SARS-CoV2-related pneumonia, and our results may be not generalizable to these patients. Similarly, our results are not generalizable to other kind of pneumonia, such as healthcare-associated pneumonia [17]. In addition, our results apply to patients not needing advanced ventilation or intubation. Finally, due to the relatively small number of NOAF occurrences in our cohort, we were unable to conduct subgroup analyses, and the multivariable analyses could not encompass all potential variables associated with NOAF.

In conclusion, C_2_HEST score represents a useful tool to predict the risk of NOAF in patients with CAP and may be used in clinical practice to identify patients with higher risk and improve preventive and treatment strategies to reduce early clinical complications.

## Data Availability

The data that support the findings of this study are available from the corresponding author upon reasonable request.
